# The Diffusion of Small-Pox

**Published:** 1905-05-13

**Authors:** 


### THE DIFFUSION OF SMALL-POX.

THE question of the diffusion of small-pox in the vicinity of special hospitals for its reception, and apparently by the agency of aerial convection, was the subject of an interesting debate at the March sessional meeting of the Royal Sanitary Institute held at Newcastle-upon-Tyne, the place where the occurrence of this kind of diffusion has most recently been pointed out. Dr. Buchanan, in his report to the Local Government Board concerning an epidemic of small-pox which prevailed in Gateshead and Felling in the summer of last year, strongly condemned the Sheriff Hill Small-pox Hospital, and declared that its use had been responsible directly or indirectly for a material portion of the epidemic. At the meeting to which we refer, this conclusion was disputed by Dr. Clayton, the medical officer of Health for Gateshead, who admitted that, out of 513 occurring cases between April 18th and June 5th, 56 occurred within half a mile of the hospital; but who declared that, subsequently to Dr. Buchanan's visit of inspection, he had been able to trace the origin by contact of all but four of them. He described the career of a tramp who was admitted into hospital in the pustular stage of the disease, and to whose previous perambulations 81 cases and six deaths were certainly attributable. He also related the case of a woman, in tracing whose contacts he discovered a family of eight, all suffering from the disease. One boy had been ill of small-pox for some time, and had shaved the tramp at a hairdresser's shop on the day before his admission. Not only had this tramp been shaven, but, by the kindness of the barber, he had been supplied with food in the house two or three days before. The shop was much resorted to, and it contained a roller towel which was used by several customers. Dr. Clayton's argument was, in effect, that the hypothesis of aerial convection has been hastily assumed as an explanation of cases in which direct infection might have been traced if it had been diligently sought for, or, at all events, if all the facts had been ascertainable; and there can be no doubt that this view may be correct in some instances, or that the idea of convection may be erroneously held to dispense with necessity for minute inquiry. But the general principle is, we think, too well established by the well-known instance of the Purfleet epidemic to allow us to regard small-pox hospitals as safe neighbours, or to permit their construction in thickly-populated localities. The true lesson to be learnt from the discussion is the benefit which the country would derive from following the example of Germany, and from so insisting upon vaccination that no small-pox hospitals would be required.

